# Comparison of 1D and 3D Models for the Estimation of Fractional Flow Reserve

**DOI:** 10.1038/s41598-018-35344-0

**Published:** 2018-11-22

**Authors:** P. J. Blanco, C. A. Bulant, L. O. Müller, G. D. Maso Talou, C. Guedes Bezerra, P. A. Lemos, R. A. Feijóo

**Affiliations:** 1 National Laboratory for Scientific Computing, LNCC/MCTIC, Av. Getúlio Vargas, 333, Petrópolis-RJ, 25651-075 Brazil; 2 Department of Interventional Cardiology, Heart Institute (InCor) and the University of São Paulo Medical School, Sao Paulo, SP 05403-904 Brazil; 3 INCT-MACC Instituto Nacional de Ciência e Tecnologia em Medicina Assistida por Computação Científica, Petrópolis, Brazil

## Abstract

In this work we propose to validate the predictive capabilities of one-dimensional (1D) blood flow models with full three-dimensional (3D) models in the context of patient-specific coronary hemodynamics in hyperemic conditions. Such conditions mimic the state of coronary circulation during the acquisition of the Fractional Flow Reserve (FFR) index. Demonstrating that 1D models accurately reproduce FFR estimates obtained with 3D models has implications in the approach to computationally estimate FFR. To this end, a sample of 20 patients was employed from which 29 3D geometries of arterial trees were constructed, 9 obtained from coronary computed tomography angiography (CCTA) and 20 from intra-vascular ultrasound (IVUS). For each 3D arterial model, a 1D counterpart was generated. The same outflow and inlet pressure boundary conditions were applied to both (3D and 1D) models. In the 1D setting, pressure losses at stenoses and bifurcations were accounted for through specific lumped models. Comparisons between 1D models (FFR_1D_) and 3D models (FFR_3D_) were performed in terms of predicted FFR value. Compared to FFR_3D_, FFR_1D_ resulted with a difference of 0.00 ± 0.03 and overall predictive capability AUC, Acc, Spe, Sen, PPV and NPV of 0.97, 0.98, 0.90, 0.99, 0.82, and 0.99, with an FFR threshold of 0.8. We conclude that inexpensive FFR_1D_ simulations can be reliably used as a surrogate of demanding FFR_3D_ computations.

## Introduction

Fractional Flow Reserve (FFR) is a hemodynamic index aimed at the quantification of the functional severity of a coronary artery stenosis. This index, which is calculated from pressure measurements and under hyperemic conditions, has been proposed and used to detect myocardial ischemia^1,2^, and has largely demonstrated excellent results as a diagnostic tool to defer patients with intermediate lesions to surgical procedures^3–5^.

Making use of valuable information regarding anatomy and vascular geometry contained in medical images, the scientific community specialized in computational models initiated a race pursuing the paradigm of noninvasive estimation of FFR through the use of computer simulations of coronary blood flow. A myriad of different approaches using image modalities such as coronary computed tomography angiography (CCTA)^6^, angiography (AX)^7^, and even optical coherence tomography (OCT)^8^, emerged. These approaches employ 3D models to estimate pressure losses in coronary vessels and thus to devise a strategy to predict patient-specific FFR. Such technology has been useful to improve diagnostic accuracy with respect to different traditional protocols taking the invasive measurement of FFR as gold standard^9–11^. It is important to remark that the use of 3D models carry several challenges, which range from detailed 3D lumen segmentation procedures and mesh generation to time-consuming numerical simulations in high performance computing facilities^12,13^.

In turn, simplified mathematical models, either based on the 1D Navier-Stokes equations in compliant vessels^14^ or based on compartmental (0D) representations^15^ have been employed to study different aspects of coronary physiology. Since these models neglect fundamental aspects of the 3D physics regarding flow across geometric singularities, specific models to account for focal pressure losses are usually employed^16–18^. However, these lumped models require the definition of specific parameters which may result in a cumbersome task. Validation of 1D models by using 3D simulations as gold standard for idealized phantoms and patient-specific arterial districts such as the cerebral arteries, the aorta and major vessels were reported elsewhere^19–22^. However, no validation was performed combining hyperemia, stenotic lesions and patient specific coronary territories, which are key ingredients in the computational estimation of FFR. Yet the use of 1D models to estimate FFR using CCTA or AX images has been recently proposed^23–26^, and also other works have employed simplified mathematical representations seeking to predict risk of ischemia noninvasively^27–29^. It is worth noting that, in the context of coronary circulation, the validation of 1D models against 3D models has only been reported in^30^, where a virtual patient population derived from a single CT scan was used. However, a comprehensive validation that accounts for large variability observed in terms of lumen geometry and hyperemic flow conditions is lacking.

The goal of this work is to demonstrate that 1D models are capable of predicting FFR (denoted FFR_1D_) with a high degree of accuracy when compared with FFR predicted by 3D models (denoted FFR_3D_), which is used as reference solution. To achieve this goal we make use of a sample of patients for which CCTA and IVUS images were available. After the generation of 3D models, the corresponding 1D centerlines representing the arterial topology were extracted. Then, 3D and 1D numerical simulations were performed under the assumption of hyperemic flow conditions and using appropriate boundary conditions. Several modeling scenarios are proposed from which two stand out: a scenario for practical use, and a best case scenario. The former scenario contains generic model parameters which need no tuning whatsoever. The later scenario represents the ultimate 1D modeling approach, in which several model parameters have been estimated to match data extracted from 3D models.

## Material and Methods

### Patient sample

The patient sample consisted of patients with known or suspected stable coronary disease who underwent multimodal evaluation with CCTA, angiography, invasive FFR and IVUS for diagnostic purposes. Enrollment criteria included: asymptomatic, atypical angina, ischemic equivalent, or effort angina, with no changes in functional status during the last month. Patients were referred for CCTA and, according to clinical indication, they were referred for invasive evaluation with cardiac catheterization and IVUS. The final inclusion depended on the presence of at least one de novo lesion (40% to 80% stenosis grade) in a major epicardial coronary artery with no previously implanted bypass graft which was amenable to FFR and IVUS interrogation, as judged by the interventionist. All patients underwent invasive FFR measurement and IVUS imaging to interrogate the target lesion, immediately one after the other, in the same procedure. While the invasive FFR measure was not disclosed at the time the present study was performed, the location of the FFR measurement was informed and used to present the comparison between model predictions in this study. The patient sample consisted of 20 patients and the demographics are presented in Table 1 and the detail of diseased vessels is given in Table 2. From the 20 patients, a total of 20 computational models were constructed from IVUS studies, while 9 were obtained from CCTA. From the 9 CCTA models, 12 major epicardial arteries were studied. The study protocol was approved by the Ethics Committee for the Analysis of Research Projects (CAPPesq) of Hospital das Clínicas Medical School of the University of São Paulo, and is in accordance with the Helsinki Declaration. The present is a retrospective study for which patients gave written informed consent to undergo the invasive procedure and to make data available for research. Authorization to access and make use of such available patient data was consented by the CAPPesq Ethics Committee.

**Table 1:** Summary of patient data, the mean ± SD, or n (%), are reported. Acronyms stand for: Body mass index (BMI), heart rate (HR), diastolic, systolic and mean pressures (DP, SP and MP).

Baseline clinical characteristic	Patient sample (*n* = 20)
Age, yrs	61 ± 10
Male	17 (85)
BMI, kg/m^2^	27.8 ± 3.5
Weight, Kg	83 ± 14
Height, cm	172 ± 10
HR, bpm	70 ± 8
SP, mmHg	114 ± 14
DP, mmHg	70 ± 11
MP, mmHg	85 ± 11
**Circulation Dominance**	
Right	18 (90)
Left	1 (5)
Co	1 (5)

**Table 2:** Summary of disease vessels, the quantity *n* (%) of each major epicardial artery is reported.

Artery	*n* (%)	CCTA *n* (%)	IVUS *n* (%)
LAD	23 (72)	8 (67)	15 (75)
LCX	5 (16)	3 (25)	2 (10)
RI	1 (3)	1 (8)	0 (0)
RCA	3 (9)	0 (0)	3 (15)

### Image Processing and Vascular Models

#### CCTA images

CCTA images were provided by two different scans: 64-row scanner (Aquilion 64, Toshiba Medical Systems, Otawara, Japan) and a 320-row scanner system (Aquilion ONE, Toshiba Medical Systems, Otawara, Japan). Previously to the scans, blood pressure and heart rate were assessed, sublingual fast acting nitrates and beta-blockers were orally administered. If the heart rate was above 70 beats per minute, metoprolol 50–100 mg orally and, if necessary, intravenous metoprolol (up to 15 mg) were administered. CCTA studies were performed with 50–100 mL of iodinated contrast (Iopamidol 370 mg iodine/mL; Bayer Schering Pharma, Berlin, Germany) injected intravenously at a rate of 5.0 mL/second. All acquisitions were ECG-triggered prospectively at 75% of the cardiac cycle, keeping the lowest possible radiation dose.

The segmentation procedure of CCTA images was carried out following the level-set approach proposed in^31^. Firstly, this amounts to extracting a region of interest, and then applying a curvature anisotropic filter^32^. The initialization of the level-set method was performed in individual vessels through a colliding front algorithm^31^. Finally, the lumen was defined through a marching cubes algorithm^33^.

#### IVUS images

IVUS images were acquired with the AtlantisTM SR Pro Imaging Catheter 40 MHz ECG-triggered and connected to an iLabTM Ultrasound Imaging System (Boston Scientific Corporation, Natick, MA, USA). The acquisition was performed with a frame rate of 30FPS and with constant velocity pullback at 0.5mm/s. IVUS images were gated as proposed in^34^ to retrieve the diastolic cardiac phase. Additionally, two orthogonal (along the cranial-caudal plane) AX films were acquired, also ECG-triggered, spanning 8 heartbeats. A specialist selected the images from the AX films at the full exhalation end-diastolic phase. The integration of the AX images and the IVUS dataset enabled a consistent time-coherent reconstruction of the vessel in 3D space. Then, the lumen area was manually segmented by a specialist using cubic splines. The transducer path was retrieved using both AX images through a biplane snakes method^35^. The recovered transducer path served to place the segmented luminal areas consistently with the acquisition time and the pullback velocity. Finally, the segmentation of side branches in the IVUS dataset was also manually performed. At a final stage, all contours were rotated around the axis described by the transducer path in order to minimize the mismatch between the projected luminal area from IVUS and the contrast observed in the AX film^36^.

#### Computational meshes

The triangular surfaces obtained from the pipeline described in the previous sections require further refinement to be suitable to perform 3D computer simulations. These meshes, obtained either from CCTA or IVUS imaging modalities, were further processed using the tools available in the VMTK library^37^ to generate volume meshes for 3D simulations.

**Figure 1:**
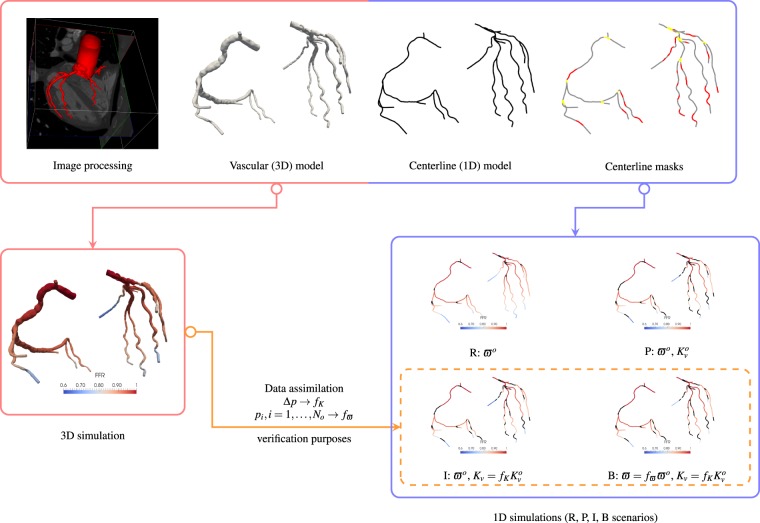
Workflow for the construction of vascular models. Image segmentation produces 3D vascular geometries which are processed to retrieve the 1D centerline geometry. Lumen area is given at each centerline point and bifurcation (yellow) and stenoses (red) masks are applied when necessary. Model scenarios are: R (*raw*): 1D model with no stenoses and known dissipation parameter *ϖ*^*o*^, P (*practical*): idem R scenario, but 1D model includes stenoses with known stenosis parameter ${K}_{v}^{o}$, I (*intermediate*): idem P scenario, but parameters *K*_*v*_ are estimated using stenosis drop pressures Δ*p* at the corresponding lesions from 3D simulations, B (*best-case*): idem I scenario, but dissipation parameter *ϖ* is estimated using outlet pressures *p*_*i*_, *I*  = 1, …, *N*_*o*_ from 3D simulations.

To construct the 1D models, the centerline of each surface mesh was computed as proposed in^38^ with a resolution of 0.05 cm between successive points. Each point of the centerline features a cross-sectional area automatically obtained from 3D model. Figure 1 features the workflow for constructing the 3D and the 1D models.

The clinically interrogated vessel was known for each patient. Moreover, the position of the invasive FFR measurement, which was informed, is denoted as ${\ell }_{{\rm{FFR}}}$ and was indicated by the specialist based on the position of the transducer according to the angiogram at the time of the invasive measurement. For comparative purposes between FFR_1D_ and FFR_3D_, the same exact arterial topology was employed in both models. According to a routine practice of invasive FFR, a minimum distance of 10 mm distally from the target lesion was observed.

In the Supplementary Material, all the models are illustrated as well as the position at which the value of the FFR was taken for comparison purposes (i.e. location ${\ell }_{{\rm{FFR}}}$).

### 3D Models

#### Mathematical Formulation

Consider a rigid vascular domain ${\rm{\Omega }}\in {{\mathbb{R}}}^{3}$, with boundary Γ whose outward unit normal vector is **n**. The boundary is decomposed into the inlet boundary Γ_*i*_, the lateral wall boundary Γ_*w*_ and the *N*_*o*_ outlets of the domain ${{\rm{\Gamma }}}_{o}^{k}$, *k* = 1, …, *N*_*o*_. Blood flow is modeled as a Newtonian fluid, therefore, the Navier-Stokes equations for incompressible flows hold

1 $$\{\begin{array}{cc}\rho \frac{{\rm{\partial }}{\bf{v}}}{{\rm{\partial }}t}+\rho ({\rm{\nabla }}{\bf{v}}){\bf{v}}+{\rm{\nabla }}p-\mu {\rm{\Delta }}{\bf{v}}={\bf{0}} & {\rm{i}}{\rm{n}}\,{\rm{\Omega }},\\ {\rm{d}}{\rm{i}}{\rm{v}}\,{\bf{v}}=0 & {\rm{i}}{\rm{n}}\,{\rm{\Omega }},\\ {\bf{v}}={\bf{0}} & {\rm{o}}{\rm{n}}\,{{\rm{\Gamma }}}_{w},\\ -p{\bf{n}}+2\mu {({\rm{\nabla }}{\bf{v}})}^{s}{\bf{n}}={P}_{{\rm{a}}{\rm{o}}}{\bf{n}} & {\rm{o}}{\rm{n}}\,{{\rm{\Gamma }}}_{i},\\ -p{\bf{n}}+2\mu {({\rm{\nabla }}{\bf{v}})}^{s}{\bf{n}}={P}_{{\rm{o}}{\rm{u}}{\rm{t}}}^{k}{\bf{n}} & {\rm{o}}{\rm{n}}\,{{\rm{\Gamma }}}_{o}^{k},\\ {\rm{w}}{\rm{i}}{\rm{t}}{\rm{h}}\,{P}_{{\rm{o}}{\rm{u}}{\rm{t}}}^{k}={P}_{{\rm{r}}{\rm{e}}{\rm{f}}}+{R}_{{\rm{o}}{\rm{u}}{\rm{t}}}^{k}{\int }_{{{\rm{\Gamma }}}_{o}^{k}}{\bf{v}}\cdot {\bf{n}}d{\rm{\Gamma }} & k=1,\,\ldots ,{N}_{o}.\end{array}$$

where **v** and *p* are the velocity and pressure fields, (⋅)^*s*^ denotes the symmetrization operation, and *ρ* and *μ* are the fluid density and dynamic viscosity, respectively. The 3D finite element strategy used to find approximate solutions to this model is described in^39^.

#### Boundary Conditions

At the inlet, a reference aortic pressure *P*_ao_ is prescribed as a Neumann boundary condition. At the outlet, resistances ${R}_{{\rm{out}}}^{k}$ are used to simulate the pressure losses in the peripheral vasculature, up to a reference venous pressure *P*_ref_. Non-invasive patient specific data, such as pulse pressure, age, weight and heart rate^40^, are used to estimate the cardiac output (CO), and then the resting coronary blood flow (RCBF) as RCBF = 0.045 × CO. Then, by using a physiological coronary flow reserve (CFR) value of 2.6 for all models, the hyperemic coronary blood flow (HCBF) is estimated as HCBF = CFR × RCBF. The HCBF, is distributed at the inlet of the major epicardial arteries following the percentages reported in Table 3. Finally, the specific flow rate through each outlet is defined using the well known Murray’s law, as described in detail in^39^. Such method defines an a priori value of flow rate, based on the classical Murray’s law, that supposedly should exit each outlet. The presence of proximal vessels with non-negligible resistance (and possibly with lesions), produces a deviation of the flow rate from that one determined by the Murray’s law, and the blood flow rates through the outlets are only known after the simulation has been finalized, and are given by

2 $${Q}_{{\rm{o}}{\rm{u}}{\rm{t}}}^{k}={\int }_{{{\rm{\Gamma }}}_{o}^{k}}{\bf{v}}\cdot {\bf{n}}d{\rm{\Gamma }}\,k=1,\,\ldots ,{N}_{o},$$

where *N*_*o*_ is the number of outlets in the model. The deviations are such that the total blood flow into the coronary tree remains invariant. These flow rates are to be prescribed later as boundary conditions for the 1D model.

**Table 3:** Percentage of the *Q*_*T*_ at the inlet of each major artery. LAD: left anterior descending, LCx: left circumflex, RCA: right coronary artery, RI: ramus intermedius.

	Circ. Dominance	LAD	LCx	RCA	RI
RI not present	Right	60	22	18	0
	Left	60	30	10	0
	Co	60	24	16	0
RI present	Right	57	10	18	15
	Left	60	15	10	15
	Co	59	10	16	15

### 1D Models

#### Mathematical Formulation

Skeletonization of Ω yields a set of centerlines, representing arterial segments, connected through a set of junctions. Centerline coordinate is denoted by *x*. The inlet boundary Γ_*i*_ is now simply denoted by the inlet point *I*, and the outlet boundaries ${{\rm{\Gamma }}}_{o}^{k}$ are denoted by *O*_*k*_, *k* = 1, …, *N*_*o*_. Given a generic centerline of size [0, *L*], the governing 1D equations are the following

3 $$\{\begin{array}{cc}\frac{{\rm{\partial }}A}{{\rm{\partial }}t}+\frac{{\rm{\partial }}Q}{{\rm{\partial }}x}=0 & {\rm{i}}{\rm{n}}\,[0,L],\\ \frac{{\rm{\partial }}Q}{{\rm{\partial }}t}+\frac{{\rm{\partial }}}{{\rm{\partial }}x}(\frac{{Q}^{2}}{A})+\frac{A}{\rho }\frac{{\rm{\partial }}P}{{\rm{\partial }}x}+\frac{2\varpi \pi \mu U}{\rho }=0 & {\rm{i}}{\rm{n}}\,[0,L],\\ P={P}_{0}+\beta (\sqrt{\frac{A}{{A}_{0}}}-1) & {\rm{i}}{\rm{n}}\,[0,L],\end{array}$$

where *Q* is the flow rate, *A* is the lumen cross-sectional area, *P* is the average pressure in the lumen cross section, $U=\frac{Q}{A}$ is the cross-sectional average velocity, *ϖ* is a parameter that characterizes the velocity profile in the 1D model, *β* is an effective stiffness which characterizes the compliance of the arterial wall, *P*_0_ is a reference external pressure for which the lumen area is *A*_0_. Since 1D models are to be compared with rigid wall 3D models, *β* is set at a high value to mimic the 1D blood flow in a quasi-rigid domain (therefore *A* ≈ *A*_0_). In practice, a per-case estimation of *β* was performed to ensure that maximum area deviations with respect to *A*_0_ are smaller than 1%.

#### Boundary Conditions

Regarding boundary conditions, the value of the pressure at the inlet in the 1D model is *P*_ao_, the same as in the 3D model. For the outflow boundary conditions, the flow rates ${Q}_{{\rm{out}}}^{k}$, *k* = 1, …, *N*_*o*_, which are given by equation (2), are prescribed. Hence, we ensure that the blood flow rate through each outlet is identical in both 3D and 1D models, guaranteeing the same blood flow distribution in the whole coronary network. In this manner, it is possible to assess the predictive capabilities of the 1D model in determining the pressure drop along the coronary tree, and in particular at lesions, by direct comparison with the 3D model, which is regarded as the reference solution.

#### Junction Models

At junctions we consider mass conservation

4 $$\sum _{i\mathrm{=1}}^{{N}_{j}}{Q}_{i}=\mathrm{0,}$$

where *N*_*j*_ is the number of converging segments. For the remaining conservation equation at junctions we consider two models. The first model is the *standard junction model* (S model), and consists of the standard assumption of conservation of total pressure, that is

5 $${P}_{1}+\frac{\rho }{2}{U}_{1}^{2}={P}_{i}+\frac{\rho }{2}{U}_{i}^{2}\,i=2,\,\ldots ,{N}_{j},$$

Note that *i* = 1 is taken as the supplier branch, i.e. the segment that provides most of the flow to the junction. The second model is denoted *dissipative junction model* (D model), and consists of the junction model proposed in^18^ which introduces pressure losses at junctions as follows

6 $${P}_{1}+\frac{\rho }{2}{U}_{1}^{2}={P}_{i}+\frac{\rho }{2}{U}_{i}^{2}+{p}_{i}^{{\rm{l}}{\rm{o}}{\rm{s}}{\rm{s}}}\,i=2,\,\ldots ,{N}_{j},$$

where

7 $${p}_{i}^{{\rm{l}}{\rm{o}}{\rm{s}}{\rm{s}}}={K}_{i}(\frac{1}{2}\rho {u}_{1}^{2}),$$

with *u*_1_ the velocity in the supplier branch. Moreover, the loss coefficient *K*_*i*_ is defined as

8 $${K}_{i}=(2{C}_{i}+\frac{{u}_{i}^{2}}{{u}_{1}^{2}}-1)\frac{{u}_{i}^{2}}{{u}_{1}^{2}},$$

with

9 $${C}_{i}=1-\frac{1}{{\lambda }_{i}{\psi }_{i}}\,\cos [\tfrac{3}{4}(\pi -{\phi }_{i})],$$

being ${\lambda }_{i}=\frac{{Q}_{i}}{{Q}_{1}}$, ${\psi }_{i}=\frac{{A}_{1}}{{A}_{i}}$ and ${\phi }_{i}=\frac{3}{4}(\pi -{\varphi }_{i})$, where *ϕ*_*i*_ = *θ*_1_ − *θ*_*i*_. Here, *Q*_*i*_, *A*_*i*_ and *θ*_*i*_ are the flow rate, the lumen area and the angle measured from the supplier branch, for branch *i*, while *Q*_1_, *A*_1_ and *θ*_1_ stand for the same quantities in the supplier branch. This model was developed for 2D bifurcations. Equations (4)-(5) or equations (4)-(6) are complemented with Riemann invariants for outgoing waves, resulting in a non-linear system of algebraic equations. For further details see^41^.

#### Stenosis Model

Stenoses are accounted for through the lumped parameter model proposed in^16^, for which the pressure drop across the constriction takes the form

10 $${\rm{\Delta }}P={K}_{v}\frac{\mu }{D}U+{K}_{t}\frac{\rho }{2}{[\frac{A}{{A}_{s}}-1]}^{2}{U}^{2}+{K}_{u}\rho {L}_{s}\frac{dU}{dt},$$

where *U* and *A* (*D* the diameter) are the velocity and lumen area in the unobstructed part of the vessel, *L*_*s*_ is the stenosis length, *A*_*s*_ is the minimum stenosis area, and *K*_*v*_, *K*_*t*_ and *K*_*u*_ are model parameters characterizing viscous, turbulent and inertial effects, respectively.

#### Numerical method

Equations (3) with appropriate boundary and coupling conditions are numerically solved using the local time stepping high-order finite volume method presented in^41^, where full details of the implementation are given.

### Automatic Stenosis Detection

Stenoses are detected in a fully automatic fashion to ensure reproducibility. A modified version of the algorithm proposed in^42^ to detect stenotic regions in centerlines was proposed and implemented. With this procedure, the values of *A*_*s*_ and *L*_*s*_ in equation (10) are characterized for each stenotic lesion. See Supplementary Material for details.

### Scenarios and Model Parameters

Common parameters for both models are *ρ* = 1.05 g/cm^3^ and *μ* = 4 cP. For the 3D model, the only fixed parameter is *P*_ref_ = 10 mmHg. For the 1D model, we considered the reference pressure *P*_0_ = 88 mmHg noting, however, that we model vessels as extremely stiff, so that the area will always be close to the reference area, even if the pressure in the vessel differs greatly from *P*_0_. Also, we take *K*_*t*_ = 1.52 and *K*_*u*_ = 1.0 as proposed in^16^. Patient-specific parameters are: *P*_ao_, *R*_out_, estimated as explained in^39^, and *K*_*v*_, which depends on the geometry of the lesion^43^, is given by

11 $${K}_{v}=32\frac{0.83{L}_{s}+3.28\sqrt{{A}_{s}}}{2\sqrt{A}}{[0.75\frac{A}{{A}_{s}}+0.25]}^{2}.$$

The parameters that play a main role in the viscous dissipation are *ϖ* and *K*_*v*_. Since the focus of this work is given to the pressure drop predictions delivered by the 1D model, then different scenarios are proposed, with baseline parameters *ϖ*^o^ = 11, as suggested in^44^ for coronary flow, and ${K}_{v}^{o}$ determined from equation (11). These scenarios are

- **Raw (R) scenario**: *ϖ*^*o*^, without stenosis models
- **Practical (P) scenario**: *ϖ*^*o*^ and ${K}_{v}^{o}$
- **Intermediate (I) scenario**: *ϖ*^*o*^ and ${K}_{v}={f}_{K}{K}_{v}^{o}$, *f*_*K*_*estimated*
- **Best-case (B) scenario**: *ϖ* = *f*_*ϖ*_*ϖ*^*o*^ and ${K}_{v}={f}_{K}{K}_{v}^{o}$, *f*_*K*_ and *f*_*ϖ*_ both *estimated*

Observe that we created the R scenario in which there are no stenoses in the models. That is, models are purely 1D, and there is no need neither to apply the stenosis detection algorithm nor to define parameters *K*_*t*_, *K*_*u*_, *K*_*v*_. In the P scenario the stenosis detection algorithm is applied, and all parameters are defined uniformly for all stenoses in all patients. In scenarios I and B, we refer to *estimated* parameters, which implies that data from 3D simulations is extracted and some parameters are identified using a Kalman filter-based data assimilation approach (see^45^ for details) to deliver the best possible match. Factor (or stenosis factor) *f*_*K*_ is estimated to define the value of *K*_*v*_ for each stenosis in each vascular network such that the pressure drop Δ*P* delivered by the stenosis in the 1D model matches the pressure drop obtained in that stenosis from the 3D model. Factor (or profile factor) *f*_*ϖ*_ is estimated to define the value of *ϖ*, which defines the velocity profile for each vascular network, such that the pressure at outlet locations in the 1D model matches the pressure obtained in these outlets from the 3D simulation. For both estimates (*f*_*K*_ and *f*_*ϖ*_), the cost functionals used are the time-averaged errors along a single cardiac cycle.

Scenarios I and B are reported because they feature the best achievable results in terms of 1D modeling. In fact, parameters in these cases are stenosis-specific (*K*_*v*_) and patient-specific (*ϖ*) and constructed to match data from 3D simulations.

These four scenarios are combined with the two junctions models: S (standard, see equation (5)) and D (dissipative, see equation (6)) for a total of eight scenarios, denoted by Y_X_, Y ∈ {R, P, I, B} and X ∈ {S, D}.

### Statistical Analysis

In order to perform the comparisons between FFR_3D_ and FFR_1D_, the solution of the 3D simulations is lumped to the centerline by slicing it with planes orthogonal to the centerline tangent direction at each centerline point and then by averaging the pressure value at each slice. Finally, the FFR_3D_ is computed by normalizing all these average values with the aortic pressure. The 1D model directly delivers the pressure value at each centerline point, so that the computation of the FFR_1D_ is straightforward. In this way, we have FFR_3D_ and FFR_1D_ for all the points along the centerlines of the coronary network for each anatomical model.

The comparison between FFR_3D_ and FFR_1D_ according to the different scenarios is realized at four relevant locations in each network. These locations correspond to major vessels (left anterior descending, circumflex, ramus intermedius and right coronary arteries) specifically in the set of points ${\ell }_{4{\rm{P}}}=\{\frac{\ell }{4},\frac{\ell }{2},\frac{3\ell }{4},\ell \}$, where $\ell$ is the total length of the vessel. In addition, and since the location of the invasive FFR measurement was informed, this is also used as a point for comparison, being denoted by ${\ell }_{{\rm{FFR}}}$.

Comparisons are reported using mean and standard deviation of the FFR values and of the discrepancies between 3D and 1D models. A Bland-Altman analysis (*m*_BA_ ± SD_BA_) is also presented. Pearson’s correlation coefficient *r*, as well as coefficients of the linear approximation FFR_1D_ = *a*FFR_3D_ + *b* are also computed. A cut-off value of FFR_3D_ = 0.8 is used to identify functional stenoses. By taking FFR_3D_ as the reference solution, we report the prevalence (Prev), and classification indexes such as the area under the receiver operating characteristic curve (AUC), accuracy (Acc), sensitivity (Sen), specificity (Spe), positive predictive value (PPV) and negative predictive value (NPV) for the FFR_1D_ computed in different scenarios.

## Results

Table 4 presents the statistical comparison between the stenotic pressure drop in the 3D model and the stenotic pressure drops as predicted by all the 1D scenarios described in Section 1.6. The statistics of the stenosis morphology defined by the automatic algorithm explained in Section 1.5 is also reported, where $\frac{{A}_{s}}{A}$ is the severity and *L*_*s*_ the stenosis length, see equations (10) and (11). Also, the Reynolds number computed in the 3D model is given as the average between the inlet and outlet Reynolds numbers. In the cases of scenarios I and B, the statistics of the value of the stenosis factor *f*_*K*_ estimated using the Kalman filter is given. Finally, in the B scenario, the statistics of the profile factor *f*_*ϖ*_ estimated using the Kalman filter is reported.

**Table 4:** Comparison of stenotic pressure drop (Δ*P*) between the 3D model and all 1D scenarios (for both junction models, D: dissipative and S: standard), in [mmHg], for all stenoses.

Junction Model	Δ*P* [mmHg] 3D	1D scenarios Δ*P* [mmHg]			B: best	Junction Model	$\frac{{{\boldsymbol{A}}}_{{\boldsymbol{s}}}}{{\boldsymbol{A}}}$	*L*_*s*_ [cm]	Re	*f*_*K*_ scen. I, B	*f*_*ϖ*_ scen. B
		R^†^: raw	P^†^: practical	I^†^: intermediate							
D	6.75 ± 9.08	5.35 ± 4.29	5.52 ± 6.58	5.25 ± 6.54	5.16 ± 6.56	D	0.50 ± 0.13	0.55 ± 0.37	185 ± 94	0.97 ± 0.51	0.74 ± 0.16
S						S					0.76 ± 0.22

Marker ^†^ indicates scenarios with *p* > 0.05 in the paired U-Test, meaning that no significant differences between Δ*P* of 1D and 3D predictions was found. $\frac{{A}_{s}}{A}$: stenosis degree, *L*_*s*_: stenosis length; Re: Reynolds number; *f*_*K*_: stenosis factor estimated by the Kalman filter; *f*_*ϖ*_: velocity factor estimated by the Kalman filter. Note that *f*_*ϖ*_ statistics are computed over *n* = 6 computational models for which at least one stenosis was detected. The remaining statistics were computed using *n* = 15 stenosis elements.

Table 5 reports the statistical analysis of the results at ${\ell }_{4{\rm{P}}}$ and ${\ell }_{{\rm{FFR}}}$ locations, summarizing the performance of the eight scenarios involving 1D models with respect to the prediction of the 3D model.

**Table 5:** Statistical results of predictive capabilities of FFR_1D_ when compared with FFR_3D_ for the different scenarios Y_X_, Y ∈ {R, P, I, B} and X ∈ {S, D}, with R: raw, P: practical, I: intermediate, B: best, S: standard junction and D: dissipative junction.

		Scenario	Linear approx.		Corr.	FFR_1D_ − FFR_3D_	Prediction value of FFR_1D_ vs. FFR_3D_					
			*a*	*b*	*r*	*m*_BA_ ± SD_BA_	AUC	Acc	Sen	Spe	PPV	NPV
Location of comparison	${\ell }_{4{\rm{P}}}$ (*n* = 156)	R_D_	0.72	0.26	0.88	0.00 ± 0.04^†^	0.97	0.98	0.80	0.99	0.89	0.99
		P_D_	0.96	0.03	0.95	0.00 ± 0.03^†^	0.97	0.98	0.90	0.99	0.82	0.99
		I_D_	0.97	0.03	0.96	0.00 ± 0.03^†^	0.97	0.98	0.90	0.99	0.82	0.99
		B_D_	0.92	0.08	0.96	0.00 ± 0.02	0.96	0.99	0.90	1.00	1.00	0.99
		R_S_	0.69	0.29	0.87	0.01 ± 0.04^†^	0.97	0.98	0.80	0.99	0.89	0.99
		P_S_	0.94	0.06	0.94	0.00 ± 0.03^†^	0.97	0.98	0.90	0.99	0.82	0.99
		I_S_	0.95	0.05	0.94	0.00 ± 0.03^†^	0.98	0.99	0.90	0.99	0.90	0.99
		B_S_	0.93	0.07	0.95	0.00 ± 0.03	0.95	0.99	0.90	1.00	1.00	0.99
	${\ell }_{{\rm{FFR}}}$ (*n* = 32)	R_D_	0.70	0.26	0.80	−0.01 ± 0.07	0.99	0.97	1.00	0.97	0.75	1.00
		P_D_	1.01	−0.03	0.92	−0.02 ± 0.05	0.99	0.97	1.00	0.97	0.75	1.00
		I_D_	1.02	−0.04	0.92	−0.02 ± 0.05	0.99	0.97	1.00	0.97	0.75	1.00
		B_D_	1.02	−0.03	0.92	−0.01 ± 0.05^†^	1.00	1.00	1.00	1.00	1.00	1.00
		R_S_	0.69	0.28	0.79	−0.01 ± 0.07^†^	0.99	0.94	0.67	0.97	0.67	0.97
		P_S_	1.00	−0.01	0.91	−0.02 ± 0.05^†^	0.99	0.94	0.67	0.97	0.67	0.97
		I_S_	1.01	−0.03	0.91	−0.02 ± 0.06^†^	0.99	0.94	0.67	0.97	0.67	0.97
		B_S_	1.04	−0.04	0.93	−0.01 ± 0.05^†^	1.00	0.97	0.67	1.00	1.00	0.97

Values of FFR compared at four locations, ${\ell }_{4{\rm{P}}}$, in the interrogated vessel as well as at the clinically relevant location for diagnosis, ${\ell }_{{\rm{FFR}}}$. Sample sizes are obtained from the 29 computational models. Prevalence of functional stenoses according to FFR_3D_: 0.06 for ${\ell }_{4{\rm{P}}}$ and 0.09 for ${\ell }_{{\rm{FFR}}}$. Linear approximation coefficients defined by *a* and *b*. *r*: Pearson’s correlation coefficient (*p *< 0.05 for all models). *m*_BA_±SD_BA_: mean and standard deviation of Bland-Altman analysis for the difference FFR_1D_−FFR_3D_. Marker ^†^ indicates correlation (*p* ≥ 0.05) between 1D and 3D models. Predicted values (AUC, Acc, Sen, Spe, PPV, NPV) computed using FFR_3D_ as gold standard and a cut-off value of FFR ≥ 0.8.

**Figure 2:**
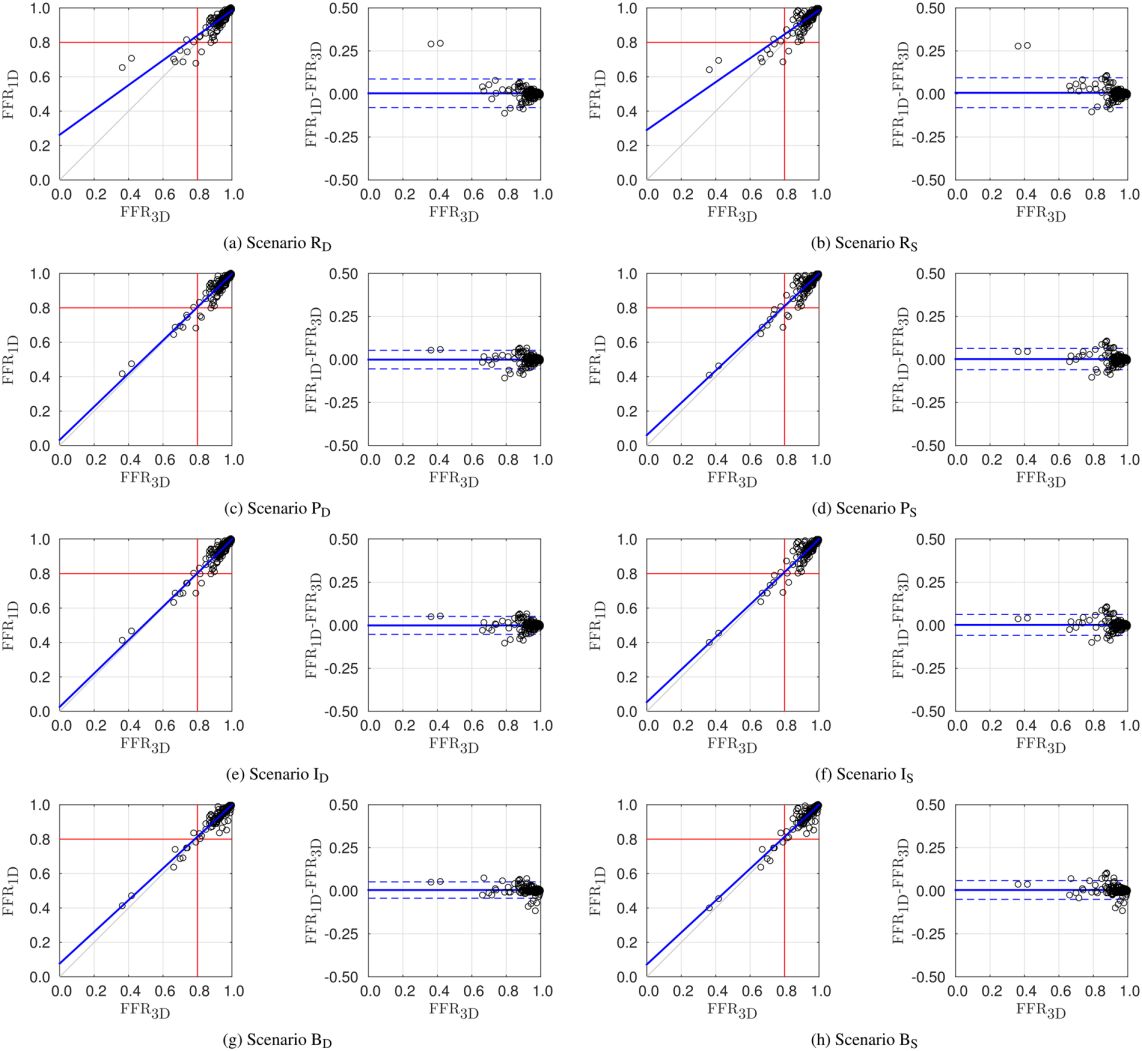
Scatter and Bland-Altman plots featuring comparison between the gold standard FFR_3D_ and FFR_1D_ for different scenarios, Y_X_, Y ∈ {R, P, I, B} and X ∈ {S, D}, with R: raw, P: practical, I: intermediate, B: best, S: standard junction and D: dissipative junction. Results correspond to four locations, ${\ell }_{4{\rm{P}}}$, in the interrogated vessels.

Figure 2 displays the scatter plot and the Bland-Altman plots for the comparison between FFR_3D_, the gold standard, and FFR_1D_ as given by the eight different scenarios.

**Figure 3:**
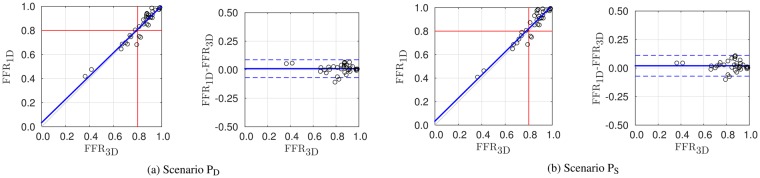
Scatter and Bland-Altman plots featuring comparison between the gold standard FFR_3D_ and FFR_1D_ for scenarios P_S_ and P_D_. Results correspond to the alternative set of points ${\hat{\ell }}_{4{\rm{P}}}$, which yields a prevalence of 0.28 (*n* = 36).

Additionally, we have taken a subset of the points used in the comparison displayed in Fig. 2 by selecting vessels for which at least one of its points featured FFR_3D_ < 0.85. This subset is denoted by ${\hat{\ell }}_{4{\rm{P}}}$. Naturally, this subset results in a relatively larger set of points with positive FFR. The results are displayed in Fig. 3 only for the practical scenarios P_S_ and P_D_.

Concerning computational requirements, the comparison is performed discarding the image processing stage. Such image and mesh processing stage is the same for both 3D and 1D models, except for the generation of the 3D volume mesh (not required for the setting of the 1D model). In terms of computational resources required by the simulations, a cluster consisting of 100 nodes with 2 x Intel Xeon X5670 2.93 GHz (6 cores), 36GB of RAM and 54 nodes with 2 x Intel Xeon E5-2660 2.20 GHz (8 cores), 64GB of RAM interconnected through Infiniband QDR, was employed. The 3D models of CCTA and IVUS contained, respectively, 2 ± 1 M and 1.6 ± 0.5 M degrees of freedom. In turn, 1D models contained 348 ± 189 and 162 ± 40 degrees of freedom, for CCTA and IVUS, respectively. The 3D simulations employed 130 ± 52 computational tasks, depending on model size, while all 1D simulations made use of 12 computational tasks. In order to provide a theoretical comparison of the speed-up provided by 1D models, we normalize the wall-clock time (WCT), that is the effective time taken for the simulation to execute one cardiac cycle, multiplying it by the number of computational tasks (CT) required, which gives what we call the normalized time (NT). This is NT = WCT × CT. Thus, per cardiac cycle, 3D simulations resulted in WCT_3D_ = 27.22 ± 22.41 hs and NT_3D_ = 3212 ± 2710 hs, while 1D simulations resulted in WCT_1D_ = 0.09 ± 0.09 hs and NT_1D_ = 1.12 ± 1.06 hs. It is worth noting that the computational times reported for the 1D model have to be considered as a worst case scenario. In fact, in order to mimic the rigid nature of the 3D domain, we use large values for *β*, resulting in very small time steps in order to satisfy the CFL condition required by our numerical scheme, which uses an explicit time discretization. Our experience suggests that using physiological values for *β* would result in near real-time 1D simulations. Moreover, previous work^39,46^ has shown that steady state 3D simulations are accurate enough for FFR prediction. In the 1D case, such conditions imply that one has to solve a simple differential-algebraic system (instead of a system of partial differential equations), with consequent further reduction in the computational cost.

## Discussion

### Comparison between FFR_1D_ and FFR_3D_

Inspecting the results reported in Table 5 at the four locations ${\ell }_{4{\rm{P}}}$, we observe that all 1D model scenarios provide excellent classification capabilities when compared to the 3D model. Moreover, almost no bias and a small standard deviation are obtained for the difference FFR_1D_ − FFR_3D_. Overall, considering the junctions as dissipative (model D), instead of standard (model S), does not bring substantial improvements neither to the correlation coefficients (*a*, *b*, *r*, *m*_BA_, SD_BA_) nor to the classification indexes (AUC, Acc, Sen, Spe, PPV, NPV). More in detail, we note that the plain 1D model (scenarios R_D_ or R_S_) provides the poorest correlation with the 3D model (coefficients of linear regression *a* = 0.72, *b* = 0.26, *r* = 0.88 for R_D_). By adding the stenosis model with a one-fits-all strategy for the parameter calibration (scenarios P_D_ or P_S_), the correlation coefficients significantly improve (*a* = 0.96, *b* = 0.03, *r* = 0.95 for P_D_). This can also be appreciated in Fig. 2, where the alignment of the point cloud around the 45 line is clear. In such plot, the correction of some outliers is also noticeable. As a consequence, the use of stenosis models is mandatory for the 1D modeling approach.

Moreover, notwithstanding the setting of stenosis-specific parameters tuned with 3D data brings some improvements, there is no considerable gain in the correlation coefficients (*a* = 0.97, *b* = 0.03, *r* = 0.96 for I_D_), while classification indexes remain invariant. Even in the best case scenario in which we further estimate the velocity profile such that 1D terminal pressures match those of 3D models, there is no relevant improvement in the model capabilities. The stenosis parameter estimated from 3D data resulted in average very close to the unit value (see Table 4) which was taken in the one-fits-all approach, and as suggested in the original contribution^16^.

Analyzing the prediction of FFR in clinically relevant locations ${\ell }_{{\rm{FFR}}}$ (see Table 5), we observe that both the correlation coefficients and the classification indexes continue to be excellent, and the AUC index grows almost to a perfect unitary value. Particularly, the only low value is obtained for the PPV, which is in part a consequence of the low prevalence sample. However, the best case scenarios (scenarios B_D_ and B_S_) demonstrate that, when properly tuned, 1D models provide an exact match with 3D models in term of diagnostic capabilities. Furthermore, at these clinically relevant locations, since in average they are more distally placed, cumulative effect of upstream junctions causes the dissipative junction (scenarios D) to outperform the standard junction (scenarios S). Therefore, our recommendation is to employ this junction model.

The relatively low prevalence of positive FFR_3D_ values in the ${\ell }_{4{\rm{P}}}$ population, equal to 0.06, might raise concern as whether this fact is being favorable to validating our working hypothesis on the ability of 1D simulations to match 3D results. In order to rule out such concern we have considered an alternative population ${\hat{\ell }}_{4{\rm{P}}}$ in which we only included the points of ${\ell }_{4{\rm{P}}}$ for vessels in which at least one of its points had a FFR_3D_ < 0.85. In that case, the number of sampled points is 36, with a prevalence of 0.28. Statistics for all scenarios are consistent between populations ${\ell }_{4{\rm{P}}}$ and ${\hat{\ell }}_{4{\rm{P}}}$. Particularly, results for scenario P_D_ (see Fig. 3) are: mean difference of 0.01 ± 0.04, significant correlation of 0.96, slope of 0.97 and intercept of 0.03 for the linear regression and overall AUC, accuracy, sensitivity, specificity, positive predictive value, and negative predictive value of 0.98, 0.92, 0.90, 0.92, 0.82, 0.96. Comparison of these results with the ones reported in Table 5 for the same scenario show that considerations about the accuracy of 1D simulations for FFR prediction remain valid for this sample population.

In a nutshell, practical scenarios making use of one-fits-all stenosis parameters are sufficient for an excellent estimation of the pressure drop occurring as predicted by 3D simulations. The use of dissipative junctions is not mandatory, although it slightly improves the capabilities of FFR_1D_, with more gains at clinically relevant locations.

### Pressure drop at lesions

It is important to stress that the comparisons presented here exclusively focus on the ability of 1D models to predict pressure drops Δ*P* in stenotic lesions. This has been achieved by setting the same flow rate boundary conditions extracted from the 3D models to the 1D counterparts, guaranteeing the consistency in the flow regime in among the models. The discrepancy in the stenotic Δ*P* estimation is also observed in Table 4. While the plain 1D model (scenarios R_D_ or R_S_) underestimates the Δ*P*, the inclusion of stenosis models yields larger Δ*P*, rendering the alignment in the correlation line seen in Fig. 2. Noteworthy, the pressure drop at junctions remains the same whether the D or the S models for the junctions are used. This is expected, as the junction model does not modify the flow rate through the vessels, since it is prescribed through each outlet, and so the pressure drop exclusively depends on the stenosis model.

On the one hand, regarding the lesion-specific parameter *K*_*v*_ estimated from 3D data (scenarios I and B), it is remarkable that, in average, it results very close to the unit value (i.e. *f*_*K*_ = 0.97 ± 0.51), close to the value chosen for the one-fits-all approach in scenario P (theoretically *f*_*K*_ = 1). On the other hand, regarding the characterization of the velocity profile given by network-specific parameter *ϖ*, the estimation using 3D data indicates that there is room for improvement (i.e. *f*_*ϖ*_ = 0.74 ± 0.16, model D) in order to improve the selection criterion for such parameter.

### Outlook for FFR_1D_

In this work we have shown that 1D models enable accurate and fast prediction of FFR when compared to the reference solution provided by 3D simulations, making such models serious candidates to be considered as surrogates of 3D models to aid the decision-making in clinical routine. As it can be appreciated from analyzing the computational cost (either wall-clock time and normalized time) required by the simulations, the clear advantage of 1D simulations is that they only require the use of workstations which are easily available in medical facilities, while 3D models make use of clusters of high performance computers in most of the cases, posing an unrealistic scenario in clinical practice. Furthermore, we highlight that 1D models do not require the exact delineation of the boundary between the lumen and the arterial wall, as 3D models do, but only an accurate characterization of the value of the lumen area. Moreover, computational meshes for 1D models are trivially defined, while generating volume meshes for 3D models can be time-consuming and in some cases technically challenging. These aspects facilitate the image processing stage, and offers promising conditions towards the full automation of methodologies using FFR_1D_.

Concerning the calibration of model parameters, the 1D simulations performed in the present work made use of the flow rates extracted from 3D models to set boundary conditions. This criterion was taken in order to keep invariant the fluid flow regime in the arterial vessels. This choice enable us to focus the comparison on the prediction of pressure loses which occur internally in the geometrical model of the coronary tree under consideration. In practice, the proposed methodology does not actually require the 3D simulations, but must be provided with a set of boundary conditions properly defined according to any given physiological criteria. In this sense, 1D simulations are fully independent of 3D simulations. For instance, flow rates can be determined from patient clinical data (age, weight, height, cuff pressure) and from coronary geometry, as proposed in^39^.

The next stage in the present line of research will regard performing an ultimate assessment of the predictive power of the FFR_1D_ in the clinical setting. This amounts to compare FFR_1D_ to invasive measures of FFR, as well as to perform sensitivity analysis and uncertainty quantification, which were out of the scope of the present contribution. Remarkably, the realization of these studies can now be tackled using this kind of simplified models, providing an effective approach to predict FFR non-invasively.

### Limitations

As in any non-invasive FFR approach, one of the limitations in the present methodology is the determination of the geometric model on top of which computational simulations are performed. In this regard, IVUS images provide a more accurate representation of the lumen in the interrogated vessel than CCTA, although CCTA geometries inform better about the network topology. The interplay between the benefits and disadvantages of each of these models was assessed elsewhere^39^, by comparing the predictions obtained from IVUS geometric models and those derived from CCTA geometric models. In this sense, the IVUS study is less prone to suffer from image artifacts such as lumen underestimation either in the presence of calcified lesions or due to intensity attenuation. Uncertainty quantification with respect to lumen segmentation has been carried out in the context of CCTA images in^47,48^, while uncertainty quantification in the FFR predictions from IVUS images has not yet been studied. However, regardless of the imaging modality (IVUS or CCTA), it is important to highlight that, once the lumen segmentation has been defined, both models the 3D and the 1D, are fed with the same surface geometry. That is, using the 3D model as the reference solution, the input for the 1D model is exact, which makes the conclusions of the present study (comparing 3D and 1D simulations) independent of the errors in the geometry definition. A limitation shared by both 3D and 1D models is the definition of boundary conditions based on power laws, which may not be accurate, especially in the presence of coronary lesions^12^. Finally, while keeping in mind that the goal of this work has been to show how to obtain 1D model results that accurately reproduce 3D model results, the validation of our framework for non-invasive FFR prediction against invasive FFR mesurements will be addressed in future works.

## Final Remarks

The results reported in this work indicate that FFR_1D_ simulations can be reliable surrogates of FFR_3D_ models to assess functional significance of coronary stenoses. Even if 1D models must be endowed with stenoses models to effectively predict pressure drops in lesions, a one-fits-all strategy to set up stenoses parameters rendered excellent predictive capabilities in terms of classification indexes when regarding the FFR_3D_ as the reference solution. Adopting such practical strategy, and adding dissipative junctions, when compared to FFR_3D_, FFR_1D_ renders a mean difference of 0.00 ± 0.03 (−0.02 ± 0.05), and overall accuracy, sensitivity, specificity, positive predictive value, and negative predictive value of 0.98, 0.90, 0.99, 0.82, and 0.99 (0.97, 1.00, 0.97, 0.75, and 1.00), respectively, to detect significant stenoses at several locations in the coronary network (at clinically relevant locations).

Remarkably, it has been possible to show that, in the best case scenario conceived by setting properly calibrated stenoses parameters, the FFR_1D_ perfectly matched the classification given by FFR_3D_.

Even if the present results have been obtained for a exploratory sample of patients, the results constitute a first validation survey to test the hypothesis that inexpensive FFR_1D_ can be reliably used instead of FFR_3D_ simulations. As a matter of fact, FFR_1D_ has the benefit of requiring substantial less time and computational resources than FFR_3D_, which makes this approach affordable in clinical routine. Having demonstrated that the FFR_1D_ concept is feasible, our next step consists in validating the proposed FFR_1D_ methodology in the clinical setting.

## Electronic supplementary material

Supplementary Material

## Data Availability

The data that support the findings of this study are available on request from the corresponding author [PJB]. The data are not publicly available due to privacy restrictions of research participants.
