# Author Correction: Comparison of 1D and 3D Models for the Estimation of Fractional Flow Reserve

**DOI:** 10.1038/s41598-018-37078-5

**Published:** 2018-12-14

**Authors:** P. J. Blanco, C. A. Bulant, L. O. Müller, G. D. Maso Talou, C. Guedes Bezerra, P. A. Lemos, R. A. Feijóo

**Affiliations:** 1 National Laboratory for Scientific Computing, LNCC/MCTIC, Av. Getúlio Vargas, 333, Petrópolis-RJ, 25651-075 Brazil; 2 Department of Interventional Cardiology, Heart Institute (InCor) and the University of São Paulo Medical School, Sao Paulo, SP 05403-904 Brazil; 3 INCT-MACC Instituto Nacional de Ciência e Tecnologia em Medicina Assistida por Computação Científica, Petrópolis, Brazil

Correction to: *Scientific Reports* 10.1038/s41598-018-35344-0, published online 22 November 2018

The original version of this Article contained a typographical error in the spelling of the author P. A. Lemos, which was incorrectly given as P. L. Lemos. This has now been corrected in the PDF and HTML versions of the Article, and in the accompanying Supplementary Material file.

