# Supplementary material for: Comparison of 1D and 3D Models for the Estimation of Fractional Flow Reserve
Source: Sci Rep. 2018 Nov 22;8:17275. doi: 10.1038/s41598-018-35344-0 (PMC6250665; doi:10.1038/s41598-018-35344-0)
Supplement: Supplementary file 1 — Supplementary Material [file 41598_2018_35344_MOESM1_ESM.pdf]

Supplementary Material: Comparison of 1D and 3D Models for the Estimation of Fractional Flow Reserve

P.J. Blanco[1,3,*], C.A. Bulant[1,3], L.O. Müller[1,3], G.D. Maso Talou[1,3], C. Guedes Bezerra[2,3], P.A. Lemos[2,3], and R.A. Feijóo[1,3]

[*]pjblanco@lncc.br
[1]National Laboratory for Scientific Computing, LNCC/MCTIC, Av. Getúlio Vargas, 333, Petrópolis-RJ, 25651-075, Brazil
[2]Department of Interventional Cardiology, Heart Institute (InCor) and the University of São Paulo Medical School, São Paulo, SP, 05403-904, Brazil
[3]INCT-MACC Instituto Nacional de Ciência e Tecnologia em Medicina Assistida por Computação Científica, Petrópolis, Brazil

ABSTRACT

In this supplementary material we explain some specificities of the procedure to create the 1D model.

1 Computational models

The 9 CCTA models are displayed in Figure 1, and the 20 IVUS models are displayed in Figure 2. The region where the invasive measurement was performed is indicated for each model and is denoted by ℓ_{FFR}.

2 1D Mesh Generation

Meshes for 1D simulations are built on top of centerline models, generated using vmtk [1]. Such centerlines are discretized with a node spacing of 0.05 cm, and each node contains, as attribute, the radius of the maximum circumscribed sphere at that point, we call this variable r_s[cm]. The cross-sectional areas can be retrieved by slicing the 3D mesh at each centerline point, the radius of the circle with equivalent area is denoted as r_a[cm].

As described in Section 2.3, junctions and stenoses can be treated with special mathematical models. Therefore, two masks are added to the centerline to mark regions inside junctions and delimiting stenotic lesions. The process to generate both masks is fully automatic to ensure reproducibility, and is described next.

2.1 Identification of Junctions

The junction mask is zero at each centerline point outside a junction region, and is greater than zero for each point p_i satisfying

$$\|\mathbf{v}(p_i) - \mathbf{v}(p_j)\| \leq r_s(p_i) + r_s(p_j), \quad (1)$$

where $\mathbf{v}(p)$ is the spatial coordinate of centerline point p, and p_j is any point of the centerline not belonging to the parent of the artery containing p_i. Additionally, a correction is performed to ensure that the ratio (in terms of r_a) between the last point of a junction mask and the first of the associated segment is less than $3/4$, which is done expanding the junction mask if necessary. This is performed to avoid numerical errors in the simulation, due to artificial discontinuities in the cross-sectional area at the inlet of each branch. The angle between branches is defined by the rising vectors of bifurcating vessels, which in the present case are given by the vectors tangent to the centerline at the first point of the centerline after the junction mask delimitation.

2.2 Identification of Stenoses

The stenosis mask assumes a non-zero value in regions of focal lesions and zero values elsewhere. This mask is generated after the junction mask, and therefore only points outside junctions are used, i.e. there are no intersection between junction and stenosis masks. We implement a modified version of the algorithm proposed in [2] to detect stenotic regions. Briefly, for each arterial segment, containing n points, the true lumen radius at each point i is defined as $r_i = r_a(p_i)$. Then, the hypothetical

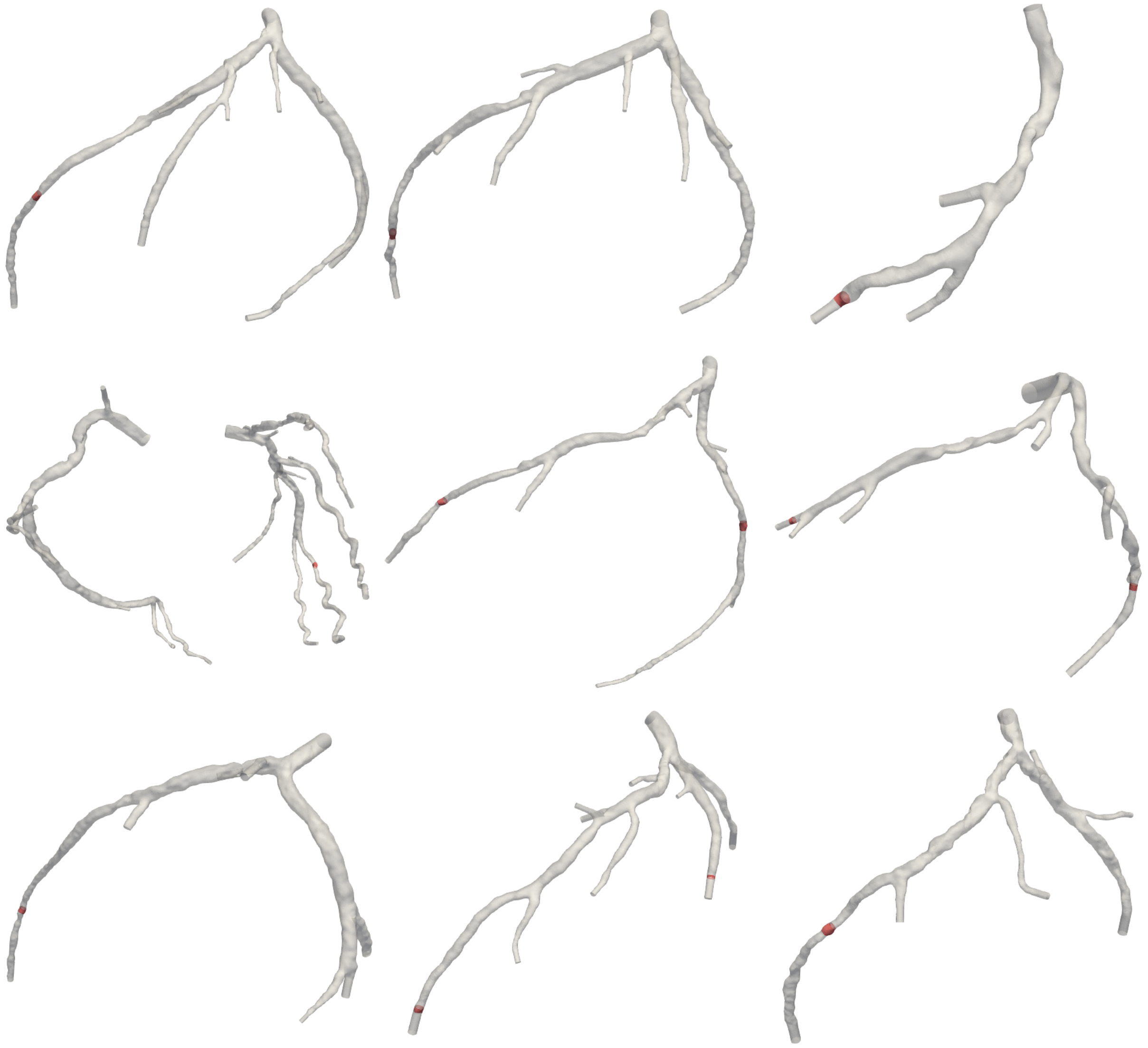

Figure 1. Computational models obtained from CCTA images. The ℓ_{FFR} region of invasive measurement of FFR is indicated with a red mark over the mesh.

"healthy" radius ($\hat{r}$) of the arterial lumen is defined by applying a robust weighted Gaussian kernel regression to the true lumen r, this is

$$\hat{r}_i = \frac{\sum_{j=1}^{n} N(j|i,\sigma_i) w_j r_j}{\sum_{j=1}^{n} N(j|i,\sigma_i) w_j} \quad \forall i \in [1,n], \tag{2}$$

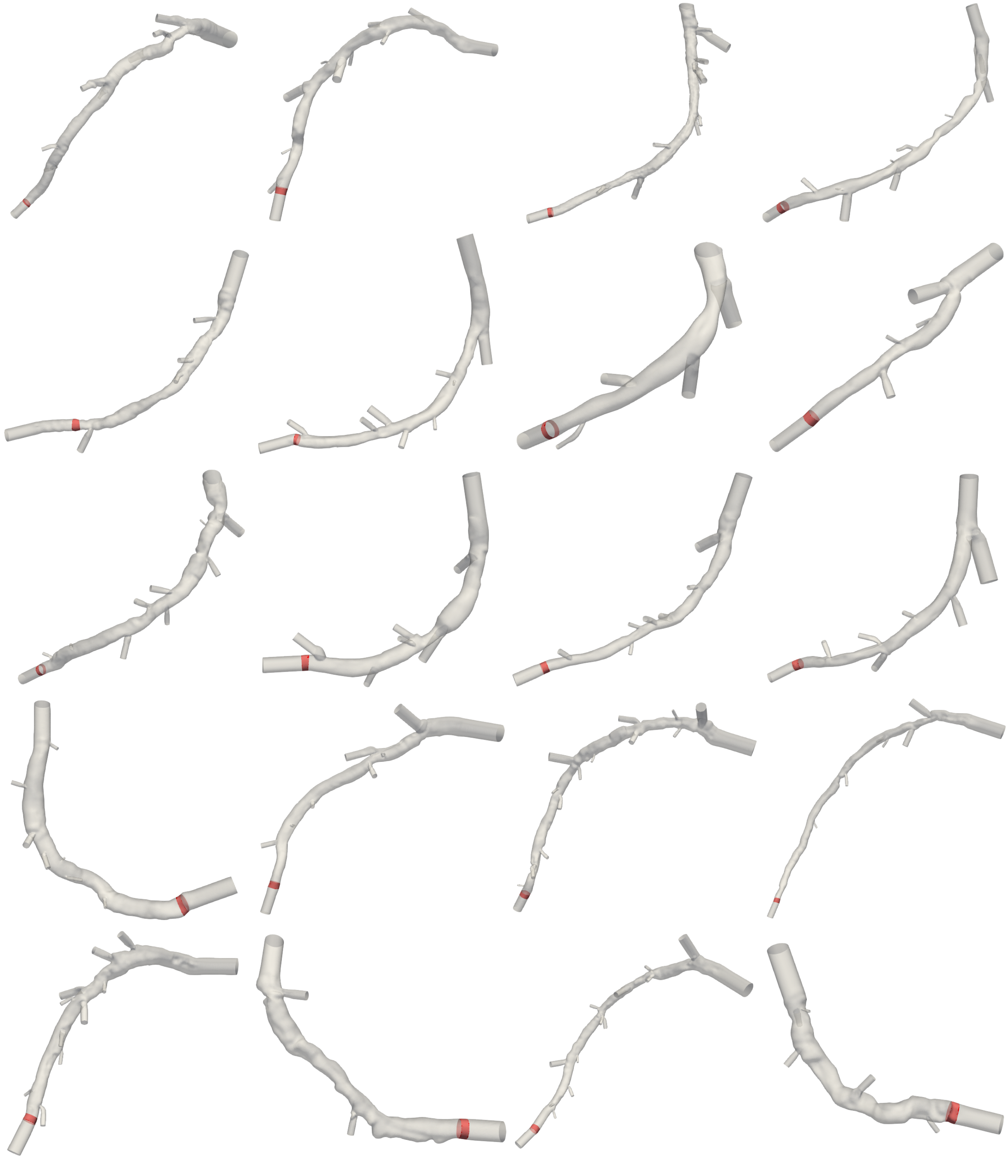

Figure 2. Computational models obtained from IVUS images. The ℓ_{FFR} region of invasive measurement of FFR is indicated with a red mark over the mesh.

where w is a weighting function and N is a Gaussian kernel, such that

$$w_i = M(r_i | r_i^{\text{MAX}}, \sigma_r), \tag{3}$$

$$r_i^{\text{MAX}} = \frac{\sum_{j=1}^{n} N(j|i, \sigma_{\max}) r_j}{\sum_{j=1}^{n} N(j|i, \sigma_{\max})}, \tag{4}$$

$$N(j|i, \sigma) = \frac{1}{\sigma\sqrt{2\pi}} e^{-\frac{(j-i)^2}{2\sigma^2}}, \tag{5}$$

$$M(r_i | r_i^{\text{MAX}}, \sigma_r) = \begin{cases} N(r_i | r_i^{\text{MAX}}, \sigma_r) & \text{if } j \leq i, \\ a & \text{otherwise} \end{cases} \tag{6}$$

Here, M is a modified kernel used to weight the radius function when its value is smaller than an approximation of the maximum radius at the location r_i^{MAX}. In the original algorithm (see [2]), $M = N$. This modification was introduced because the original algorithm attenuates radius values greater than the estimation of r_i^{MAX}. Also, when the algorithm was developed in [2], it was not clear the physical scale for which radius and vessel length were being considered, which is crucial for the definition of the parameters. Therefore, in this work, the functions representing the true lumen radius r and the arterial intrinsic length are re-sampled in 100 points and normalized in the range $[0, 1]$ prior to the computation of $\hat{r}$. Note that an abuse of notation is used when calling the Gaussian Kernel function in the arterial length space $N(i|j, \cdot)$, i and j represent the length from the arterial ostium to the points with index i and j. After computing $\hat{r}$, a postprocessing is performed to interpolate $\hat{r}$ in the original points of the centerline and in the correct range of radii, i.e. a denormalization step. The following parameters were chosen on a trial and error basis, $\sigma_i = 0.08, \sigma_r = 0.1, \sigma_{\text{MAX}} = 1, a = 10$.

The percentage area of stenosis is defined as

$$\Theta_i = 1 - \left(\frac{r_i}{\hat{r}_i}\right)^2, \tag{7}$$

and stenosis regions are detected using two threshold parameters, $\mathit{\Theta}_1, \mathit{\Theta}_2$. A stenosis is defined between two point p_i, p_j satisfying

- $\Theta_i \geq \mathit{\Theta}_1$ and $\Theta_j \geq \mathit{\Theta}_1$,
- $\Theta_{i-1} < \mathit{\Theta}_1$ and $\Theta_{j+1} < \mathit{\Theta}_1$,
- $\Theta_k \geq \mathit{\Theta}_1, \forall k \in [i, j]$,
- $\exists k \in [i, j]$ such that $\Theta_k \geq \mathit{\Theta}_2$.

The following parameters were chosen on a trial and error basis, $\mathit{\Theta}_1 = 0.1$ and $\mathit{\Theta}_2 = 0.6$.

The final 1D mesh is constructed using the centerline, by removing all points with junction mask different from zero. If a stenosis model is employed, points with stenosis mask different from zero are truncated and the arterial segment is split into two computational domains (pre- and post-lesion), connected by the lumped parameter model for the stenosis. Each arterial segment represents a one-dimensional domain, discretized using a regular mesh, i.e. constant Δx. Second order Lagrange polynomials are used to interpolate the lumen radius at centerline nodes. Gauss-Lobatto quadrature nodes are used in the numerical integration within each computational cell resulting from the spatial discretization. The mismatch between computational nodes and centerline nodes requires a post-processing of the simulation result, in which the solution is quadratically interpolated into the centerline nodes. For centerline points that are masked as stenosis points, and therefore not considered as 1D domains, the pressure is linearly interpolated between upstream and downstream results.

References

1. The vascular modeling toolkit website. URL `www.vmtk.org`.

2. Shahzad, R., Kirişli, H., Metz, C., Tang, H., Schaap, M., van Vliet, L., Niessen, W., and van Walsum, T. Automatic segmentation, detection and quantification of coronary artery stenoses on CTA. *The International Journal of Cardiovascular Imaging*, 29(8):1847–1859, December 2013. ISSN 1569-5794, 1573-0743. doi: 10.1007/s10554-013-0271-1. URL http://link.springer.com/10.1007/s10554-013-0271-1.
